# When Antipsychotics Fail and Carbamazepine Heals: A Case of Hallucinatory Psychosis Revealing a Partial Epilepsy

**DOI:** 10.1192/j.eurpsy.2026.11085

**Published:** 2026-07-31

**Authors:** W. Abid, M. Kessentini, I. Chaari, N. Halouani, L. Aribi, N. Messedi, J. Aloulou

**Affiliations:** Psychiatric department “B”, Hedi Chaker University Hospital, Sfax, Tunisia

## Abstract

**Introduction:**

Epilepsy, can manifest primarily with psychiatric symptoms, sometimes in the absence of overt seizures. These symptoms may include hallucinations, or delusional thinking, which can resemble closely schizophrenia.

**Objectives:**

We aims to call attention to psychiatric symptoms as a potential manifestations of partial epilepsy

**Methods:**

We report a case of a woman,with no personal history of epilepsy, initially diagnosed with resistant schizophrenia who experienced a resolution of symptoms after introduction of Carbamazepine, raising the hypothesis of an undiagnosed epilepsy.

**Results:**

The case is of a 49-year-old female, unmarried, with no personal history of epilepsy. She had no family psychiatric history. She was admitted to our psychiatric unit for management of persistent auditory hallucinations with command content, evolving over several years. She describes the hallucinations as hearing many voices talking together at the same time, inside her head.

Prior to hospitalisation, the patient received numerous antipsychotic treatments. She exhibited no clinical improvement. A diagnosis of treatment-resistant schizophrenia was confirmed, and she was referred to our unit for clozapine initiation.

As part of the pre-treatment assessment, several investigations were conducted. Brain imaging revealed bilateral fronto-parieto-temporal calcified subdural hematomas, while the electroencephalogram (EEG) showed no epileptiform activity.

As his length of stay in the hospital extended, there were increasingly frequent occasions when she became extremely agitated. She would hit her own head and state that she wanted to die.

Later she would apologize for her behavior. Following most of these episodes, she revealed that she had experienced auditory hallucinations during them. These hallucinations were including commands such as “bang your head against the wall” or “ kill yourself ».

A trial of clozapine was initiated and cautiously titrated, but it led to a worsening of hallucinations. The resistance to antipsychotics and the topography of the cerebral lesion, supported the hypothesis of a frontal or temporal lobe epilepsy with psychiatric presentation. A therapeutic trial of carbamazépine was conducted at a dose of 11 mg/kg/day.

The response was rapid and dramatic. The auditory hallucinations disappeared, the patient regained behavioral and emotional stability. The diagnosis of partial epilepsy was retained.

**Conclusions:**

This case highlights the importance of considering epileptic disorders as part of the differential diagnosis in cases of persistent psychosis unresponsive to antipsychotics. Even in the absence of overt seizures or abnormal EEG findings, psychiatric symptoms may represent the sole manifestation of a hidden epileptic process, particularly when brain lesions are present.

**Disclosure of Interest:**

None Declared

